# Local renin-angiotensin system in the thyroid – myth or fact?

**DOI:** 10.1186/1756-6614-6-S2-A57

**Published:** 2013-04-05

**Authors:** Mariusz Stasiołek

**Affiliations:** 1 Department of Endocrinology and Metabolic Diseases, Polish Mother’s Memorial Hospital - Research Institute, Lodz, Poland

## 

Beside the basic control system associated with the hypothalamus-hypophysis-thyroid axis and thyrotropin, a growing evidence suggests that thyroid function is influenced by a vast spectrum of locally produced factors. Renin-angiotensin system (RAS) is considered as one of the most powerful and effective regulatory systems in humans. In the “classical” cascade of RAS, angiotensinogen is enzymatically conversed to the most active element of RAS – angiotensin (Ang) II. Ang II exerts its effects via two main receptors: AT1R and AT2R. The understanding of RAS biological function was significantly changed by the discovery of new enzymatic cascades leading to the production of molecules with different than Ang II functional properties – for instance Ang (1-7), molecule reacting with its specific receptor – Mas. Beside the growing complexity of RAS-structure, diverse expression patterns of RAS components in various tissues formed a basis for a concept of “local RAS” in many organs including brain and immune system. It was shown that many organ-specific effects of hormonal factors, including the influence of thyroid hormones on cardiovascular system, were dependent on the modulation of local RAS components. However, very little is known about the role of RAS in the control of thyroid function. In our immunohistochemical analysis of operative specimens we observed for the first time expression of RAS components in human thyroid. Interestingly, although our recently published findings suggest a direct regulatory influence of thyroid function on the phenotype and activity of immune cells in humans, no studies regarding the influence of human RAS on the course of such common inflammatory process as thyroiditis are available. Flow Cytometry analyses performed in our laboratory on thyroid fine needle aspiration biopsy material allowed us to assess quantitatively and phenotypically the immune cell populations of local thyroid blood. The comparison of results obtained with paired peripheral blood and local thyroid blood samples suggested differences in the structure of antigen presenting cell populations, e.g. monocytes, known to be the main source of RAS components in the immune system. Interestingly, RAS positive immune cells have been shown to correlate with clinical course of various inflammatory and malignant diseases.

In conclusion, better knowledge about local RAS in the thyroid seems to be of great importance for the understanding of both: thyroid physiology and pathological processes associated with inflammatory and malignant disorders of this gland.

